# Vitamin D and Vitamin D Analogs as Adjuncts to Field Therapy Treatments for Actinic Keratoses: Current Research and Future Approaches

**DOI:** 10.1155/2021/9920558

**Published:** 2021-06-19

**Authors:** Zafer Sattouf, Steven J. Repas, Jeffrey B. Travers, Craig A. Rohan

**Affiliations:** ^1^Department of Pharmacology and Toxicology, Wright State University, 3640 Colonel Glenn Hwy, Dayton, Ohio 45435, USA; ^2^Department of Dermatology, Wright State University, Dayton, Ohio 45435, USA; ^3^Dayton Veterans Administration Medical Center, Dayton, Ohio, USA

## Abstract

Actinic keratoses (AK), also known as solar keratoses, are precancerous hyperkeratotic papules caused by long-term exposure to ultraviolet radiation. Management of AK prior to progression to cutaneous malignancy represents an important window of intervention. This is important on a population level, given the high incidence, morbidity, financial costs, and the low but measurable risk of mortality from cutaneous neoplasia. Treatments for AK have been refined for many years with significant progress over the past decade. Those recent advancements lead to questions about current treatment paradigms and the role of harnessing the immune system in field therapies. Recent studies suggest a key interplay between vitamin D and cancer immunity; in particular, the systemic and/or topical vitamin D analogs can augment field therapies used for severe actinic damage. In this review, we will examine the literature supporting the use of vitamin D-directed therapies to improve field therapy approaches. An enhanced understanding of these recent concepts with a focus on mechanisms is important in the optimized management of AK. These mechanisms will be critical in guiding whether selected populations, including those with immunosuppression, heritable cancer syndromes, and other risk factors for skin cancer, can benefit from these new concepts with vitamin D analogs and whether the approaches will be as effective in these populations as in immunocompetent patients.

## 1. Introduction

Actinic keratoses (AK) have the potential for malignant degeneration to squamous cell carcinoma (SCC), and that risk of malignant degeneration is increased in patients with a variety of susceptibility factors including immunosuppression, Fitzpatrick phototypes, and genetic factors [1, 2]. While the AK lesions might be unsightly or minimally symptomatic, interventions are primarily intended to mitigate the risk of progression to in situ or invasive SCC [3]. Long-standing therapies have included physical modalities such as cryotherapy with liquid nitrogen, dermabrasion, chemical peels, or curettage [4]. Areas with numerous AK lesions are termed confluent AK or field cancerization. Treatments to these entire zones of skin, termed field therapies, are used to ameliorate the risk to the entire area of skin, including normal-appearing skin adjacent to the visible AK. Field therapy options include an array of topical therapies including 5-fluorouracil, fractional laser resurfacing, chemical peels, or topical photodynamic therapy (PDT) [5]. Though overall more effective than serially treating individual lesions, field therapies suffer from multiple side effects including pain and irritation. Moreover, the effects of field therapies such as topical PDT and even 5-fluorouracil only last several years [6]. Hence, there is a need in dermatooncology for adjunctive therapies that can improve the effectiveness of field therapies. As proposed in 2002 by van den Bemd, the use of vitamin D to treat skin cancers showed promise when used topically as monotherapy in 2009 by Seckin and colleagues [7, 8]. The use of vitamin D agonists was further delineated in 2017 by Cunningham and colleagues with the expansion of the role of thymic stromal lymphopoietin (thymic stromal lymphopoietin role was first proposed by Demehri et al. in 2012) [9, 10]. The goal of this review is to update new concepts in combining vitamin D agonists with field therapies for actinic damage.

## 2. Vitamin D and Vitamin D Analogs

Vitamin D is a vital prohormone that is obtained both from the diet and from production in epidermal keratinocytes via a reaction that requires ultraviolet (UV) radiation [11]. Vitamin D undergoes two hydroxylation reactions in the liver and then the kidneys to form the biologically active form 1,25-hydroxyvitamin D or calcitriol [12]. Vitamin D plays a key role in calcium-phosphorus homeostasis. Along with well-known effects on bone development, calcium likewise has many other effects on the immune system and the skin.

Completely independent of the calcium-mediated actions, significant additional research has also demonstrated many roles in the skin for vitamin D itself, including immune surveillance of cutaneous neoplasia along with regulation of cellular differentiation and proliferation [13, 14]. Topical application of vitamin D analogs has been investigated as monotherapy for the treatment of field cancerization including actinic keratoses [8]. Topical vitamin D analogs have also been used as adjuncts to other field therapies, most prominently, with 5-fluorouracil [9].

Despite this emerging evidence on the role of systemic and topical vitamin D in ameliorating the risk of skin cancer, there are many remaining questions about vitamin D's optimal utility to decrease the longitudinal risk for skin cancer across multiple population groups (immunocompetent, immunocompromised, those with severe dermatoheliosis, and those with genetic, heritable conditions associated with increased malignancy risk). Notably, systemic vitamin D supplementation with oral or parenteral (Stoss therapy) formulations has not been investigated significantly as an adjunct to a number of current field therapy regimens.

## 3. Vitamin D, AK, and Dermatoporosis

While ultraviolet light is essential for the synthesis of vitamin D in the skin, it is also associated with nonmelanoma skin cancer (NMSC) development. Studies have shown that certain vitamin D receptor (VDR) polymorphisms are associated with a higher likelihood of developing AK [15]. These studies have encouraged many researchers to study vitamin D and its association with skin cancer; however, studies on this relationship have resulted in mixed and potentially inconsistent findings. Some studies have suggested that lower vitamin D levels increase the risk of skin cancer, while others have shown that increased levels can also increase the risk of skin cancer [16–18]. While vitamin D has a photoprotective effect on keratinocytes, studies have shown that this protection may be dose-dependent [19–21]. It is important to note that high doses of systemic vitamin D can be associated with hypercalcemic effects on the human body and although it has a wide therapeutic index, this fear has limited its use in clinical trials to further assess its systemic use as an anticancer agent. One cross-sectional study examined the status of vitamin D in patients with AK and demonstrated higher levels of serum 25(OH)D in patients with AK compared to healthy patients. This study suggested that while higher vitamin D levels correlate with higher numbers of AK, it does not increase the risk itself [22].

Patients with severe sun damage (dermatoheliosis) have been hypothesized to suboptimally synthesize vitamin D in their sun-damaged skin. This is despite their significant sun exposure, including at the wavelengths needed to produce vitamin D in the skin. This phenomenon has been termed “dermatoporosis” and a correlation between dermatoporosis and osteoporosis from insufficient skin production of vitamin D has been made [23]. This raises a number of key questions. First, whether systemic or topical vitamin D options have equivalent ability to modify the risk of neoplasia. Second, whether vitamin D's effectiveness as an adjuvant to field therapies is achieved by restoring vitamin D levels in areas of sun-damaged skin that are functionally deficient or is the effectiveness dependent on locally supratherapeutic amounts of vitamin D achieved through local, topical application of the vitamin D analogs. Another potentially important area is the discovery of novel noncalcemic vitamin D analogs which could be used in this area [13].

## 4. Vitamin D Mechanism of Action

Calcitriol and other agonists mediate their effects by interacting with the vitamin D receptor (VDR). Binding with VDR promotes heterodimerization with the retinoid X receptor, leading to translocation of this complex into the nucleus where it regulates the transcription of target genes [14, 24–27]. It is interesting that vitamin D is both produced in the skin and exerts its effects on the skin via VDR. VDR transcription rate depends on the availability of VDR agonists and antagonists and can also be influenced by single-nucleotide polymorphisms, which play a role in the etiology of NMSC [28–33]. In the skin, VDR expression can fluctuate based on the pathology of skin conditions. For instance, a knockout VDR mouse model showed an increased risk of developing skin cancer, which suggests VDR potentially functioning as a tumor suppressor [34].

In addition to its genomic activity, the VDR can additionally cause a rapid response via nongenomic, membrane-bound mechanisms, in which VDR is associated with flask-shaped invaginations in the cellular plasma membrane, called caveolae [27]. VDR studies have shown that calcitriol has a similar binding affinity to this plasma membrane-bound VDR relative to that seen with nuclear VDR. The activation of this membrane-bound, caveolae-associated VDR activates numerous rapid signal transduction pathways and second messenger systems, including phospholipase C and phospholipase A_2_, G protein-coupled receptors, phophatidylinositol-3′-kinase (PI3K), or protein kinase C. This nongenomic activity also leads to opening of calcium and chloride channels in the cell membrane [35]. The downstream effect of this membrane-bound receptor activation facilitates crosstalk inside the cell, eventually leading to changes in gene expression similar to those seen with the genomic response of VDR [27]. In fact, in vivo studies by Dixon et al. reported for the first time that this rapid-response pathway of membrane-bound VDR can indeed have photoprotective effects on UVR-induced sunburn cells [36].

In vitro studies have indicated that calcitriol exerts both antiproliferation and prodifferentiation roles on keratinocytes in a VDR-dependent manner [37]. The antiproliferation effect is mediated by inhibition of G1/S checkpoint enzymes, upregulation of inhibitors of CDKs, inhibition of cyclin D1, and upregulation of p27 and p21 [38–40]. Indirect mechanisms of 1,25(OH)_2_D_3_ antiproliferation effects also include upregulation of TGF-*β* and downregulation of EGFR found in normal keratinocytes [41–43]. As for the prodifferentiation effects, 1, 25(OH)_2_D_3_ has been shown to play an important role in the differentiation of keratinocytes from epidermal precursor cells, as well as macrophage differentiation from myelopoietic progenitor cells [37, 44]. In addition, mutations of proteins involved in both the synthesis and the catabolism of vitamin D were found to be of importance in many cancers, including SCC [45]. Additional studies on mice have reported that VDR knockout mice exposed to oral 7, 12-dimethylbenzanthracene (DMBA) had increased sensitivity to develop chemically induced skin tumors [46]. These findings, among many others, suggest an important role for vitamin D in skin cancer pathogenesis and treatment [47].

In addition to the antiproliferative and prodifferentiative effects of calcitriol on cells, calcitriol has a number of immunological effects. Calcitriol plays a role in innate immunity via increasing the expression of the human antimicrobial peptides cathelicidin and defensin *β*_2_. This increase in cathelicidin levels causes a more intense activity of phagocytic macrophages in lesional skin, such as those found in psoriasis [48–50].

## 5. Vitamin D Immunomodulatory Action

When applied topically on epithelial cells, calcitriol and its analogs have been shown to induce the expression of the cytokine thymic stromal lymphopoietin (TSLP) [51]. TSLP and IL-7 are both cytokines that are similar in their structures [52]. The receptors for TSLP and IL-7 can together form a heterodimer that is expressed on CD11c + dendritic cells [52]. TSLP binding to this receptor can induce thymus and activation-related chemokine (TARC, also known as CCL17) and macrophage-derived chemokine (MDC, also known as CCL22), resulting in a Th2-enriched inflammation [52–54]. In addition, TSLP inhibits Th1 and Th17 differentiation, which has been suggested to be associated with the reduction of psoriatic plaques [55]. A study by Sato-Deguchi and colleagues reported that vitamin D analogs induce TSLP and cathelicidin, both of which result in the suppression of IL-12/23 p40, IL-*α*, IL-1*β*, and TNF-*α*, thereby improving psoriatic plaques [55].

The immunomodulatory roles of TSLP in inflammation and cancer pathogenesis mentioned previously have become an interest for many researchers and pharmaceutical companies. Several studies have reported that TSLP can promote neoplastic growth of breast and pancreatic tissues in animal models [56–58]. Alternatively, TSLP actions have demonstrated a protective role in the carcinogenesis of skin neoplasms [59]. The study by Piazza et al. showed that this protective role was mediated by the direct signaling of TSLP on CD4+ and CD8+ T-cells that reside in the skin, resulting in an inflammatory response that can inhibit cancer development [59]. On a molecular level, activation of T-cells by TSLP was shown to inhibit the growth of *β*-catenin-dependent skin tumors. Furthermore, removing this TSLP-mediated response was shown to significantly change the inflammatory response and promote neoplastic growth in the skin [59].

The findings of an antitumoral effect of TSLP in the skin are further supported by Demehri and colleagues [9]. They found that TSLP upregulation in the skin mediated a lasting tumor rejection reaction via CD4+ T-cell response in a Th2 inflammatory pathway. This finding suggests that this antitumor response protects the skin from UV- and chemically induced neoplasms. CD4+ T-cells that were responsive to TSLP action were found to be both required and sufficient to mediate these preclinical effects of TSLP. It was also found that TSLP overexpression in the skin resulted in tumorigenesis resistance, thus confirming the tumor-suppressive effects of TSLP in the skin.

Cunningham et al. examined the efficacy of topical TSLP induction via calcipotriol in preventing skin cancer development in murine skin that was chemically treated with DMBA and tetradecanoylphorbol-13-acetate (TPA) [10, 60]. They found that calcipotriol application to the skin induced TSLP expression and resulted in a delay in developing skin cancer compared to no treatment. It was also found that calcipotriol did not have this cancer-protective effect in animal models lacking the TSLP receptor, thus clearly demonstrating the importance of TSLP in mediating this cancer immunity effect of vitamin D analogs. Even short pulses of calcipotriol application on DMBA-TPA-treated skin resulted in a transient increase in TSLP levels and exerted a long-lasting antitumor effect [10].

This TSLP upregulation of T-cell function may have implications in how vitamin D functions in immunocompromised patients (including organ transplant recipients at high risk of morbidity and mortality from NMSC) and patients with heritable, genetic cancer syndromes including those with altered DNA mismatch repair capability. One study investigated the use of topical calcipotriol cream as monotherapy or in combination with all-trans retinoic acid cream in renal transplant patients with actinic keratosis [61]. This study found that neither treatment was effective in treating actinic keratosis; however, these results cannot be generalized to the immunocompetent population. A study by Seckin et al. on immunocompetent patients with AK treated with calcipotriol as monotherapy has shown supportive results for the efficacious use of this treatment [8]. However, the absence of inflammation at treatment sites and the small number of participants in this study make the statistical significance unreliable.

## 6. Vitamin D plus 5-FU Combination Treatment for Actinic Keratosis

The promising findings of Seckin et al. prompted the use of 0.005% calcipotriol in combination with 5% 5-FU for 4 days versus vaseline for 4 days in a randomized, double-blinded clinical trial on human patients as field therapy for AK [10]. This study by Cunningham and colleagues demonstrated that this combination treatment resulted in the accumulation of lymphocytes at the sites of AK. The erythema at the site of treatment was found to be associated with various immunological reactions, including lymphocyte exocytosis, epidermal spongiosis, and dyskeratosis, resulting in tumor rejection in the skin. The majority of these lymphocytes were CD4+ T-cells, further emphasizing the antitumor role of TSLP found by Demehri et al. [9]. The inflammatory reactions seen in the combination treatment showed a significant lesional epidermis rejection pattern that was specific to the actinically damaged sites, without involving the adjacent normal tissue. It is suggested that the TSLP, HLA class II, and cellular stress signals from the cytotoxic effects of chemotherapy with 5-FU interact synergistically to mediate the immunotherapeutic effects of this combination treatment.

When comparing the combination treatment versus 5-FU alone, it is suggested that the natural killer group 2D (NKG2D) synergizes with TSLP in the combination treatment and causes suppression of cancer development [10]. This synergistic reaction does not occur when using 5-FU alone. The findings by Cunningham et al. indicated for the first time the efficacy of combination treatment of 5-FU plus calcipotriol in AK treatment. It was shown that a twice daily, 4-day application of 5-FU plus calcipotriol caused a mean reduction of 87.8% in the number of AK on the face, while 5-FU plus vaseline caused a mean reduction of 26.3%. The superior efficacy of this combination treatment was evident on other anatomical sites, including the scalp and upper extremities (see Table 1) [10]. These findings further emphasize the role of CD4+ T-cells in mediating antitumor immunity via TSLP induction. However, it is important to note that the applicability of these findings is limited to patients with competent immune systems and cannot be generalized as yet to all patients with AK.

The efficacy of 5-FU plus calcipotriol combination treatment is further highlighted by the decreased side effects seen in the treatment group in the trial using such short-term exposures. This is in contrast to side effects seen in 5-FU monotherapy for actinic keratosis which typically use much longer treatment times [62]. In addition, this combination treatment appears superior to imiquimod monotherapy as the latter only activates the innate immune system and lacks the antigen specificity provided by the HLA class II and MICB expression seen in lesional keratinocytes treated with 5-FU plus calcipotriol. This latter finding is of great importance because of the role of HLA class II and MICB in their presentation of tumor antigens. However, future studies comparing combination therapies to current monotherapy field therapy options are needed.

## 7. Vitamin D and Skin Cancer Prevention

One of the limitations of the study by Cunningham et al. [10] was focusing on the elimination of AK with the combination treatment without studying the long-term potential of preventing skin cancer development. In fact, this limitation is shared by many of the currently used treatments for AK, including photodynamic therapy, cryosurgery, imiquimod, diclofenac, and ingenol mebutate. The only currently used topical treatment that has shown a potential to prevent skin cancer in the long term was 5-FU. However, even this benefit of 5-FU treatment was limited to 2 years after treatment [63].

The long-term protection from skin cancer development in patients with AK treated with the combination of 5-FU plus calcitriol was investigated in a randomized clinical trial by Rosenberg et al. [55]. Evidence of epidermal CD4+ and CD8+ tissue-resident memory T-cells and adaptive immune response activation were found in skin treated with this combination, further confirming previous results. The majority of these cells were CD69+ and CD103+, confirming their tissue-resident memory T-cell status. In contrast to the findings of 1-year skin cancer protection by 5-FU monotherapy, the study by Rosenberg et al. found that the 4-day course of calcipotriol plus 5-FU immunotherapy was successful in preventing SCC development on the face and scalp up to 3 years following treatment [63, 64]. Eliminating precancerous lesions and yielding long-term cancer prevention potential via immunotherapeutic agents is a novel concept that has broad implications in the treatment and prevention of skin cancer and possibly even solid tumors.

## 8. Vitamin D with Other Topicals for Field Therapies and PDT

With other pharmaceuticals used to treat AK/SCC producing a significant side effect profile, the use of more combination therapies is being tested [65], one of which is the combination of topical vitamin D analogs with several FDA-approved topicals. These combinations include vitamin D with photodynamic therapy, imiquimod, ingenol mebutate, and diclofenac sodium (see Tables 2 and 3).

### 8.1. Imiquimod

Imiquimod is an immunomodulatory agent commonly used in the treatment of viral and nonviral illnesses [66]. While the mechanism of action is not definitively known, it is believed to bind to toll-like receptor 7 (TLR-7). This binding activates a directed innate immune response via IFN-*α*, TNF-*α*, IL-12, and other proinflammatory cytokines directed against intracellular pathogens and tumors [67]. In addition to acute innate immune response induction, imiquimod also influences memory T-cells and humoral immunity that creates a sustained effect on viral pathogens and tumors until they are fully eradicated [68]. The use of imiquimod against AK has been well documented as efficacious [69]. The immunomodulatory effect of imiquimod is highly effective at decreasing the amount of AK in patients with very limited amounts of recurrence [70, 71]. Significant side effects are common, which limit the frequency and concentration of dosing [72]. There have also been questions about the variability of expression of TLR7 between patients as some patients have a minimal clinical response to treatment courses.

Imiquimod has been used in conjunction with PDT in several clinical trials. Those treated with the combination showed higher rates of complete and partial clearance of AK [73]. These findings provide support for the concept that dual field therapies could be more efficacious than individual therapies. Moreover, although not reported in the literature, a trial combining a vitamin D analog, like calcipotriol, with imiquimod to see if a similar synergy is elucidated would be of significant interest.

### 8.2. Ingenol Mebutate

Ingenol mebutate has complex and incompletely understood dual mechanisms [82]. Although recent label changes and safety concerns by both the European Medicines Agency (EMA) and the US FDA have raised concerns about the long-term safety of this medicine, it remains one of the field therapies that may show enhancement with a vitamin D adjunct [83]. Ingenol mebutate causes mitochondrial membrane instability and subsequent apoptosis of tumor cells. This first mechanism shows an effect within hours of application. The exact mechanism causing this instability is unknown [84]. The second mechanism involves recruitment of neutrophils to the apoptotic cells, which mediates antibody-dependent cellular cytotoxicity [85]. This is a delayed response and involves reactive oxygen species released from neutrophils. Ingenol mebutate has been shown to successfully treat AK and prevent the progression to SCC [86, 87]. It is also well documented to have a superior tolerability profile when compared to other topicals used to treat AK [88, 89].

Similar to imiquimod, ingenol mebutate has been used in combination with PDT in several clinical trials [90]. These studies concluded that the combined effect of PDT and ingenol mebutate was superior to these therapies individually, indicating a potential synergistic effect [91]. Patients also reported high satisfaction and tolerability with the combinatory therapy [92]. These promising findings warrant further clinical studies and new combinations. Currently, there are no published studies showing the effects of ingenol mebutate when paired with vitamin D analogs.

### 8.3. Diclofenac Sodium

Diclofenac sodium is a member of the nonsteroidal anti-inflammatory drug family [93]. Diclofenac sodium is a nonselective competitive inhibitor of cyclooxygenases (COX-1 and COX-2) within the prostaglandin synthesis pathway [94]. AK and SCC have been shown to have upregulated COX-2 expression with subsequent prostaglandin E_2_ accumulation [95]. Downregulation of COX-2 byproducts may explain the beneficial use of topical diclofenac sodium in treating both AK and SCC [96]. Further studies have indicated a potential induction of apoptosis within precancerous and cancerous cells as a proposed mechanism of diclofenac sodium [97]. Other mechanisms for the anticarcinogenic effects of COX inhibition could include augmenting antitumoral immunity. Clinically, topical diclofenac sodium is one of the most easily tolerated field treatments for AK [98, 99].

The majority of studies that combine the effects of diclofenac and vitamin D are documented in mice. It has been found that there is a significant reduction in tumor load and overall tumor prevention in mice with the synergistic effects of calcipotriol and diclofenac sodium [100]. Other preclinical studies have reported the superiority of this combination versus placebo in decreasing tumor size [101]. While these animal studies show a great deal of potential, there are a number of gaps within the literature [100]. First, there are no published human studies of this combination against AK/SCC to show side effect profiles, safety profiles, and efficacy [102]. Further, the exact mechanism of the synergy seen in these animal studies has yet to be elucidated and warrants further investigation.

### 8.4. Photodynamic Therapy

As described in previous reviews, PDT remains an important field therapy option and treatment of several dermatological cancers [103]. PDT is conducted by injecting different types of sensitizers such as 5-aminolevulnic acid (5-ALA) or methyl aminolevulinate (MAL) into the bloodstream or by more commonly applying them topically [104]. These sensitizers are taken up by cells and subsequently catalyze the production of phototoxic hemoglobin metabolites, in the case of both ALA and MAL, protoporphyrin IX. Protoporphyrin IX has been shown to have an antiangiogenesis effect on the microvasculature in highly proliferating cells [105, 106]. Furthermore, these photosensitizers induce platelet aggregation and other inflammatory cytokines in these cells, creating an innate immune response [107]. Next, a very specific wavelength of light is focused on target areas with confluent AK [108]. The focused light interacts with the protoporphyrin IX creating reactive oxygen species (ROS) within cells; these induce apoptosis in a very precise area of the skin [109, 110]. PDT has been well documented in its efficacy and safety profile in treating AK and SCC alike with AK clearance one year after treatment up to 47% [111, 112]. PDT use in AK/SCC treatment is generally well tolerated by patients [113].

The concomitant use of PDT with calcipotriol and other vitamin D analogs has been examined [73, 114–116]. Preclinical studies using murine BCC and SCC demonstrated the synergistic effects of vitamin D agonists to “precondition” the tumors before PDT [114–116]. These important preclinical studies have not as yet been fully translated into human studies. However, the addition of topical calcipotriol has been shown to significantly improve AK clearance [117] compared to MAL or 5-ALA alone [118]. Patients also tolerated PDT with vitamin D analogs well [119]. Mechanistic studies suggest that the addition of calcipotriol increases coproporphyrinogen oxidase, a heme breakdown enzyme, which creates additional ROS within cancerous cells, increasing apoptosis in these cells [120].

## 9. Future Recommendations

There are several therapeutic questions remaining. These include the efficacy and tolerability of vitamin D analogs to treat AK on extremities and nonfacial skin. While 5-FU plus calcipotriol combination treatment showed AK reduction and SCC protection on the face and scalp, no similar significant differences were noted for field therapy treatments on the extremities. This limitation can be addressed by future studies using the same treatment but potentially for a longer period of time, enough to induce a higher degree of erythema and adaptive immune response via T-cell activation in AK lesions. In addition, 5-FU plus calcipotriol showed no potential benefit in preventing BCC, further supporting the immune-based mechanism of this therapy [64]. Mutations in AK and SCC are similar in their nature of antigen presentation, whereas BCC mutations involve the hedgehog signaling pathway [121].

A second therapeutic gap is designing treatments, which would be effective in high-risk populations, especially those with compromised immune systems. It is important to note that combination therapy of 5-FU with calcipotriol appears dependent on the adaptive immune response and infiltration of T-cells in immunocompetent patients. This important finding can theoretically limit the use of this novel treatment in immunocompromised and organ transplant patients. MAL-PDT, cryotherapy, and 5-FU are currently the treatments of choice for AK in transplant patients [122]. A clinical trial of calcipotriene monotherapy, all-trans retinoic acid monotherapy, or a combination of both has been previously studied in renal transplant patients. Unfortunately, none of these treatments had a marked reduction in the number of AK lesions [61]. However, with 5-FU as one of the standard treatments for transplant patients with AK, the novel 5-FU plus calcipotriol combination might potentially be of higher benefit to these patients. Future research is especially important for these immunocompromised patients and other high-risk patients, whose risk of progression to SCC and the subsequent risk of more serious complications of SCC is increased. This population may require combination treatments that are less reliant on the patient's own immune system, for example, fractional laser resurfacing, photodynamic therapy with aminolevulinic acid, or chemical peels.

A final knowledge and therapeutic gap concerns the use of topical vs systemic vitamin D analogs in combinatorial therapy. A potential advantage of oral or injected (Stoss) vitamin D is its low cost. Future studies could help identify whether subsequent systemic repletion, independent of any comorbid vitamin D deficiency, could decrease the risk for actinic neoplasia.

## Figures and Tables

**Table 1:** Summary of results of the clinical trial of 5-FU plus calcipotriol versus 5-FU plus vaseline [10].

		5-FU plus calcipotriol (%)	5-FU plus vaseline (%)
Mean reduction in the number of actinic keratoses	Face	87.8	26.3
	Scalp	76.4	5.7
	RUE	68.8	9.6
	LUE	76	16.3

Complete clearance of AK on the face	27		0

Partial clearance of AK	Face	80	0
	Scalp	56	0
	RUE	30	4
	LUE	56	3

% of participants who had reduction of AK counts on all treated anatomical sites	Face	100	80
	Scalp	100	71
	RUE	100	65
	LUE	100	77

RUE: right upper extremity; LUE: left upper extremity.

**Table 2:** Comparison between current treatments of AK.

Clinical trial/studies	Primary outcomes/findings
5% 5-FU + glycolic acid (GA) vs. GA alone [74]	(1) Combination treatment cleared 92% of AK on the face at 6 months vs. 20% with GA alone

% 5-FU vs. 30% trichloroacetic acid peel vs. CO_2_ laser resurfacing [75]	(1) 83% to 92% AK reduction in treatment groups vs. control (no treatment) (2) Lower incidence of NMSC in comparison with control (3) No significant difference in reduction of AK or NMSC protection among the treatment groups (4) Limited by small participants number

5% 5-FU vs. CO_2_ laser resurfacing (LA) [76]	(1) CO_2_ laser resurfacing resulted in less AK recurrence (2) Recurrence rates for LA were 21.7%, 21.7%, and 25.9% at 3, 6, and 12 months after treatment (3) Recurrence rates for FU were 61.5%, 57.7%, and 60.0% at 3, 6, and 12 months after treatment (4) Side effects more frequent in the laser resurfacing group, especially hypopigmentation and erythema

5% 5-FU vs. cryotherapy vs. imiquimod [77]	(1) Total clearance rates of AK were 68% for cryotherapy, 85% for imiquimod, and 96% for FU (2) Higher rates of recurrence at 12 months seen in the FU and cryotherapy-treated group (3) Imiquimod was judged to have the best cosmetic outcomes

5% 5-FU vs. imiquimod [78]	(1) 5-FU was more effective than imiquimod in exposing subclinical AK, reducing AK counts at 24 weeks (94% vs. 66%), achieving complete clearance at 24 weeks (84% vs. 24%) (2) Erythema as a side effect was higher in the 5-FU arm

0.5% 5-FU vs. placebo [79]	(1) Mean AK count reduction at 4 weeks was 62.4% in the 5-FU group vs. 28.8% in the vehicle group (2) Complete clearance was 16.7% with FU and 0% in the vehicle group

0.5% 5-FU vs. ALA PDT (blue light) vs. ALA PDT (laser light) [80]	(1) 5-FU was as equivalent to blue-PDT in achieving >75% AK clearance (2) Cumulative clearance rate at 4 weeks after treatment for 5-FU, blue-PDT, and PDL-PDT was 79%, 80%, and 50%, respectively (3) PDT using ALA plus laser light was the least effective among the treatment arms (4) Both PDT treatment arms had lower side effect profile than 5-FU

Long-term efficacy of 5% 5-FU [81]	(1) At 6 months after treatment, fewer AK were found in 5-FU treated patients compared to control (no treatment) (2) Higher complete AK clearance in 5-FU vs. control (38% vs. 17% at 6 months) (3) Fewer spot treatments at 6-month intervals and in between study visits were reported in the 5-FU treated patients (4) No difference in the number of hypertrophic AK between the two groups

5% 5-FU prevention of skin cancer [63]	(1) At 1 year after treatment, a 75% risk reduction of developing squamous cell carcinoma was seen in patients treated with 5-FU vs. vehicle cream (2) No significant effect was seen in the development of basal cell carcinoma

**Table 3:** Brief meta-analysis of FDA-approved topical therapies combined with topical vitamin D analogs in the treatment of AK.

Compound	Study	Primary outcomes/findings
Imiquimod	(1) Bhatta et al. [102] (2) Baneerjee et al. [69] (3) Befon et al. [123]	(1) Enhanced efficacy against invasive squamous cell carcinoma (2) Increased clearance rate of actinic keratoses and change in lesion count (3) Effectively cleared both clinical and subclinical actinic keratoses

Ingenol mebutate	(1) Rosen et al. [82] (2) Gras [124] (3) Anderson et al. [125]	(1) Treatment for only 2-3 days resulting in rapid lesion necrosis of actinic keratoses (2) Significant reduction of head and nonhead actinic keratoses in a short duration of treatment (3) Significant complete clearance rate and partial clearance rate in nonfacial actinic keratoses

Diclofenac sodium	(1) Pommergaard et al. [100] (2) Ulrich et al. [95] (3) Martin et al. [98]	(1) Significant reduction of tumor size (2) Significant field cancerization treatment of actinic keratoses (3) Significant long-term clearance of actinic keratoses

Photodynamic therapy	(1) Fu et al. [111] (2) Neittaanmaki-Perttu et al. [126] (3) Heppt et al. [73]	(1) Significant increased complete clearance rate of grade II-III actinic keratoses lesions (2) Complete histological clearance with increased patient preference due to low adverse effects (3) Increased clearance rates compared to monotherapy alone

## Abbreviations

AK: Actinic keratosis
SCC: Squamous cell carcinoma
NMSC: Nonmelanoma skin cancer
VDR: Vitamin D receptor
TSLP: Thymic stromal lymphopoietin
COX: Cyclooxygenase
5-FU: 5-Fluorouracil
PDT: Photodynamic therapy
TLR-7: Toll-like receptor 7
DMBA: 7,12-Dimethylbenzanthracene
NKG2D: Natural killer group 2D.
